# Recorded Early Imaging Responses After Transarterial Chemoembolization Plus Lenvatinib Versus Transarterial Chemoembolization Without Documented Lenvatinib for Unresectable Hepatocellular Carcinoma: A Vietnamese Cohort Study

**DOI:** 10.3390/jcm15176839

**Published:** 2026-09-03

**Authors:** Vu Le Minh, Nguyen Van Hung, Pham The Anh, Pham Tuan Anh, Pham Minh Thong

**Affiliations:** 1 Department of Diagnostic Imaging, Hanoi Medical University, 1 Ton That Tung Street, Kim Lien Ward, Hanoi 116177, Vietnam; vuleminh156@gmail.com (V.L.M.); phamminhthong@hmu.edu.vn (P.M.T.); 2 Diagnostic Imaging Center, Vietnam National Cancer Hospital (K Hospital), 30 Cau Buou Street, Thanh Liet Ward, Hanoi 100000, Vietnam; 3 Department of Diagnostic Imaging, Thai Binh University of Medicine and Pharmacy, 373 Ly Bon Street, Tran Lam Ward, Hung Yen Province 06122, Vietnam; 4 Department of Hepatobiliary and Pancreatic Surgery, Vietnam National Cancer Hospital (K Hospital), 30 Cau Buou Street, Thanh Liet Ward, Hanoi 100000, Vietnam; theanhbenhvienk@gmail.com; 5 Department of Optimal Clinical Care, Vietnam National Cancer Hospital (K Hospital), 30 Cau Buou Street, Thanh Liet Ward, Hanoi 100000, Vietnam; phamtuananh@hmu.edu.vn

**Keywords:** hepatocellular carcinoma, transarterial chemoembolization, lenvatinib, imaging response, missing data, real-world evidence, Vietnam

## Abstract

**Background/Objectives:** Vietnamese evidence on transarterial chemoembolization (TACE) plus lenvatinib for unresectable hepatocellular carcinoma (HCC) remains limited. We examined recorded early imaging responses and uncertainty from missing outcomes, exposure classification, and confounding. **Methods:** This single-center secondary cohort included all eligible available records during a shared post-approval period (21 February 2024–19 August 2025). The endpoint was a documented complete or partial response according to the Response Evaluation Criteria in Solid Tumors version 1.1 (RECIST v1.1) at the first recorded post-index computed tomography (CT) or magnetic resonance imaging (MRI), an ascertainment-dependent surrogate rather than a standardized clinical-trial objective response rate. We reported risk differences (RDs), risk ratios (RRs), odds ratios (ORs), 95% confidence intervals (CIs), nonparametric bounds, tipping-point analysis, sequential models, and a comparator co-therapy audit. **Results:** Among 108 patients (23 combination; 85 comparator), recorded responses occurred in 17/23 (73.9%) and 30/85 (35.3%): RD, 38.6 percentage points (95% CI, 18.0–59.2); RR, 2.09 (95% CI, 1.44–3.05); and OR, 5.19 (95% CI, 1.85–14.57). With 6 and 34 undocumented outcomes, response-difference bounds ranged from −1.4 to 64.7 percentage points. Reclassifying 12 undocumented comparator outcomes as responses made Fisher’s *p*-value exceed 0.05; reclassifying 33 eliminated the point-estimate advantage when all six undocumented combination outcomes were nonresponses. The expanded complete-case estimate was attenuated (adjusted OR, 1.97; 95% CI, 0.45–8.60). Excluding five comparator records with explicit systemic co-therapy yielded an adjusted OR of 5.49 (95% CI, 1.93–15.61). Safety incidence and survival were not estimable. **Conclusions:** Recorded responses differed between strategies, but missingness, imaging ascertainment, treatment-selection confounding, and incompletely characterized comparator exposure prevented identification of a comparative treatment effect. This study identifies registry and implementation gaps and generates a hypothesis for prospective modified RECIST (mRECIST)-based evaluation.

## 1. Introduction

Hepatocellular carcinoma (HCC) remains a major cause of cancer-related morbidity and mortality, with a particularly high burden in East and Southeast Asia [1]. Treatment selection integrates tumor stage, hepatic reserve, performance status, technical resectability, prior treatment, and access to locoregional and systemic therapies [2,3,4,5]. Transarterial chemoembolization (TACE) remains an established option for many patients with intermediate-stage or otherwise unresectable liver-confined disease, although this population is clinically heterogeneous and repeated TACE may compromise hepatic reserve.

TACE induces ischemia and tumor necrosis, but residual viable tissue may become hypoxic and upregulate vascular endothelial growth factor (VEGF), fibroblast growth factor (FGF), and other proangiogenic pathways [6,7,8]. Lenvatinib inhibits VEGF receptors 1–3, FGF receptors 1–4, RET, KIT, and platelet-derived growth factor receptor alpha, providing a biologically plausible complement to TACE [9]. In the phase III REFLECT trial, lenvatinib was noninferior to sorafenib for overall survival and achieved a higher tumor response rate in unresectable HCC [10].

Evidence for TACE–lenvatinib combinations has expanded. The randomized LAUNCH trial showed improved outcomes with TACE plus lenvatinib compared with lenvatinib alone in advanced HCC [11]. Retrospective comparative studies in intermediate-stage or unresectable HCC have also reported higher response proportions and, in some cohorts, longer progression-free or overall survival than TACE alone [12,13,14,15,16]. However, most evidence comes from East Asian centers outside Vietnam and remains heterogeneous in response criteria, treatment sequencing, comparator definition, imaging follow-up, and control of confounding.

We evaluated recorded early imaging responses after TACE plus lenvatinib versus TACE without documented lenvatinib in a Vietnamese institutional cohort. The principal scientific issue was not statistical significance alone, but whether the observed difference was identified despite differential outcome availability, incomplete treatment histories, and treatment-selection confounding. We therefore treated the endpoint as an ascertainment-dependent recorded response, quantified missing-outcome bounds and tipping points, and framed the study as a hypothesis-generating assessment of both clinical practice and registry completeness rather than as a causal comparative-effectiveness estimate.

## 2. Materials and Methods

### 2.1. Study Design, Setting, and Period

This was a single-center secondary comparative cohort analysis of two pre-existing institutional data sources at the Diagnostic Imaging Center, Vietnam National Cancer Hospital (K Hospital), Hanoi, Vietnam: a prospectively maintained research database for patients receiving TACE plus lenvatinib and a separate comparator database containing patients who underwent TACE without documented lenvatinib. The source files included combination-group index procedures from 11 October 2022 to 12 May 2026 and comparator procedures from 21 February 2024 to 19 August 2025. To reduce secular bias and ensure that all reported index treatments occurred after ethics approval, the analysis was restricted to the shared post-approval period from 21 February 2024 to 19 August 2025. Records outside this period were not analyzed.

### 2.2. Participants and Exposure Classification

The source-protocol eligibility criteria included age ≥18 years; HCC diagnosed histologically or according to accepted Vietnamese imaging and biomarker criteria [17]; disease considered unsuitable for resection, transplantation, or curative ablation; at least one measurable lesion; Eastern Cooperative Oncology Group performance status 0–2; Child–Pugh class A hepatic function; and adequate hematologic, hepatic, and renal function. Major exclusions were another active malignancy, active infection, severe uncontrolled comorbidity, brain metastasis or spinal cord compression, known hypersensitivity to study treatment, or inability to undergo TACE. Several eligibility and baseline fields were incompletely retained in the comparator source; therefore, all source-protocol criteria could not be independently reverified for every comparator record.

Records required a valid index TACE date and a database-level exposure classification. Combination exposure required explicit documentation of lenvatinib or “Len” in clinical or procedure text. Records documenting sorafenib, prior immunotherapy without documented lenvatinib, or no identifiable lenvatinib exposure were excluded from the combination group. The comparator was defined by absence of documented lenvatinib in the available record, not by verified absence of all systemic therapy. All comparator free-text fields were manually screened for explicit mentions of immunotherapy, targeted therapy, sorafenib, lenvatinib, or other systemic anticancer treatment. Because complete medication histories and treatment timing were not retained, the comparison is described as TACE plus verified lenvatinib versus TACE without documented lenvatinib; it is not presented as a strict comparison with confirmed TACE monotherapy.

### 2.3. Record Inclusion and Study Size

The analysis included all eligible available records within the two database-defined sampling frames during the shared post-approval period. This was not a population census and does not imply population representativeness. No separate a priori sample-size calculation was performed for this secondary analysis. The combination source contained 46 candidate records; 5 were excluded because lenvatinib exposure could not be verified and 18 were outside the shared analysis period, leaving 23 combination records. All 85 comparator records had index TACE dates within the analysis period. The final cohort comprised 108 patients.

### 2.4. Index Treatment, Treatment Strategy, and Follow-Up Timeline

The index treatment was the first TACE procedure used to anchor database inclusion during the shared analysis period. In the combination source, the intended lenvatinib starting dose was 8 mg once daily for body weight < 60 kg and 12 mg once daily for body weight ≥ 60 kg, with interruption or dose reduction according to toxicity and clinical judgment; TACE was planned 1–5 days after lenvatinib initiation. Conventional TACE used an iodized oil–chemotherapy emulsion followed by embolic material, whereas drug-eluting bead TACE used chemotherapy-loaded microspheres. Selective or superselective catheterization was performed when technically feasible. “Hybrid/combined TACE” was retained as recorded because procedural components were not standardized sufficiently in the extract for further subclassification. *Product-level manufacturer information for lenvatinib, iodized oil, chemotherapeutic agents, embolic materials, catheters, and microspheres was not retained in the secondary analytic extract and therefore could not be reported reliably*.

The target contrast was the observed initial management strategy, not the isolated biological effect of one TACE procedure. Actual lenvatinib initiation and continuation dates, interruptions, dose intensity, repeat TACE before first imaging, and the exact index-to-imaging interval were not uniformly recoverable. Repeat TACE was clinically determined by residual viable tumor, hepatic function, performance status, and multidisciplinary review. Because first recorded imaging could occur after additional treatment, the endpoint should be interpreted as responses recorded during the early management strategy rather than responses attributable solely to the index procedure.

### 2.5. Recorded Imaging Endpoint and Other Outcomes

The endpoint was a documented complete response (CR) or partial response (PR), assessed according to the Response Evaluation Criteria in Solid Tumors version 1.1 (RECIST v1.1), at the first recorded post-index computed tomography (CT) or magnetic resonance imaging (MRI) assessment [18]. The source protocol specified imaging at approximately 8-week intervals, but exact intervals, modality by group, reader identity, reader blinding, and adjudication were not consistently retained. Stable disease (SD), progressive disease (PD), and undocumented responses were tabulated when represented. SD was not explicitly represented in the analytic extract, and reasons for missing responses were unavailable. Source images were not centrally reread. Accordingly, the endpoint is an ascertainment-dependent surrogate—documented RECIST v1.1 response at first recorded imaging—rather than a standardized, centrally adjudicated clinical-trial objective response rate (ORR). Recorded categories were not relabeled as modified RECIST (mRECIST) responses because viable enhancing tumor was not independently reassessed [4,5,19].

The secondary descriptive variables were initial TACE technique, number of documented TACE sessions, postembolization syndrome, free-text lenvatinib-related adverse-event entries, grade ≥3 event entries, treatment-related-death entries, and availability of progression and death dates. Adverse events were intended to be graded according to Common Terminology Criteria for Adverse Events version 5.0 [20]. Because safety fields and longitudinal laboratory monitoring were incomplete and non-harmonized across sources, adverse-event incidence was not estimable. Comparative progression-free and overall survival were not analyzed because progression, death, last-contact, and censoring dates were incomplete and differentially recorded.

### 2.6. Data Collection and Quality Control

Data were collected using the standardized case report form developed for the source protocol and maintained in two Microsoft Excel workbooks (Microsoft Corporation, Redmond, WA, USA; source software version not retained). Variables included age; index and imaging dates; viral-hepatitis markers; alpha-fetoprotein (AFP); albumin; bilirubin; tumor-burden score; maximum tumor diameter; portal vein tumor thrombosis (PVTT); TACE technique and number of sessions; response category; progression and death dates; postembolization syndrome; and free-text treatment and adverse-event descriptions. The albumin–bilirubin (ALBI) score was calculated as (0.66 × log10 bilirubin [μmol/L]) − (0.085 × albumin [g/L]), with grades assigned using established cutoffs [21].

Repeated procedure rows were consolidated into one patient-level record; dates were standardized; exposure text was manually verified; and duplicate and implausible-date checks were performed. Patients were classified as response-available when CR, PR, SD, or PD was recorded. Separate indicators described missing baseline prognostic variables and missing responses. Exact treatment timelines, imaging intervals, reader characteristics, and reasons for missing responses were not uniformly recoverable. The analytic dataset was de-identified.

### 2.7. Causal and Measurement Framework

Before interpreting adjusted models, we specified a causal and measurement framework (Supplementary Figure S1). Potential treatment-selection confounders included calendar time and access, multidisciplinary judgment, tumor burden, PVTT, hepatic reserve/ALBI, performance status, AFP, prior therapy, and anticipated treatment tolerance. Initial TACE technique and repeat treatment could reflect both clinical selection and treatment intensity. The source database and documentation process could influence exposure classification, covariate availability, imaging timing, and whether a response was recorded. This framework was used to separate observed recorded responses from underlying tumor responses and to avoid treating limited regression adjustment as adequate causal control.

### 2.8. Statistical Analysis

Continuous variables were summarized as median and interquartile range (IQR); categorical variables were summarized as number and percentage with explicit denominators. Baseline *p*-values were not emphasized. For binary characteristics, absolute standardized differences (ASDs) were calculated using the pooled Bernoulli variance; values > 0.10 were used only as a descriptive flag for imbalance. Means and standard deviations were not available for continuous variables, so median differences were reported instead of standardized mean differences.

The principal descriptive estimate was the proportion of all records with a documented CR or PR. Undocumented outcomes remained unknown; they were not asserted to be biological nonresponses. We reported the absolute risk difference (RD), risk ratio (RR), odds ratio (OR), and 95% confidence intervals (CIs). To make outcome missingness the central inferential issue, we calculated nonparametric worst-case bounds by allowing each undocumented binary response to be either a response or a nonresponse. A deterministic tipping-point analysis held all six undocumented combination outcomes as nonresponses and sequentially reclassified 0–34 undocumented comparator outcomes as responses. We identified the number required for the two-sided Fisher’s exact *p*-value to exceed 0.05, for the Wald OR confidence interval to include 1, and for the point estimate of the RD to cross 0. A response-evaluable analysis was reported only as a selected-subset description.

Sequential logistic models were retained as limited descriptive adjustments: Model 1 included age (per 10-year increase) and treatment year; Model 2 additionally included initial TACE technique (conventional versus nonconventional); and an exploratory complete-case model additionally included AFP > 400 ng/mL and continuous ALBI score. PVTT was not entered because only two events were recorded, both in the combination group, creating sparse-data and separation concerns. Tumor-burden score and maximum tumor diameter were not entered because they were almost entirely unavailable among patients without a recorded response. Stratum-specific treatment estimates using the TACE technique were not presented because the hybrid stratum contained 7 patients versus 1 patient and differential outcome missingness would make such estimates unstable. A restricted exposure analysis excluded comparator records with explicit systemic co-therapy. Missing baseline covariates were not multiply imputed because source-dependent co-missingness, sparse auxiliary information, and likely missing-not-at-random processes made a credible missing-at-random model untenable; transparent bounds were preferred to model-based precision unsupported by the data. Analyses used Python 3.11 (Python Software Foundation, Beaverton, OR, USA), SciPy (exact version not retained), and statsmodels (exact version not retained). Reporting followed the Strengthening the Reporting of Observational Studies in Epidemiology (STROBE) recommendations [22].

### 2.9. Ethics

The study protocol was approved by the Institutional Review Board for Biomedical Research of Hanoi Medical University (IRB-VN01.001/IRB00003121/FWA 00004148; approval certificate No. 843/GCN-HĐĐĐNCYSH-ĐHYHN; approved 4 May 2023). The reported comparative cohort included only index treatments after the approval date. The study was conducted in accordance with the Declaration of Helsinki and relevant institutional requirements. The analytic dataset contained no directly identifying information. Informed-consent requirements and any applicable waivers were governed by the IRB-approved protocol and institutional requirements.

## 3. Results

### 3.1. Record Inclusion and Baseline Imbalance

**Figure 1:**
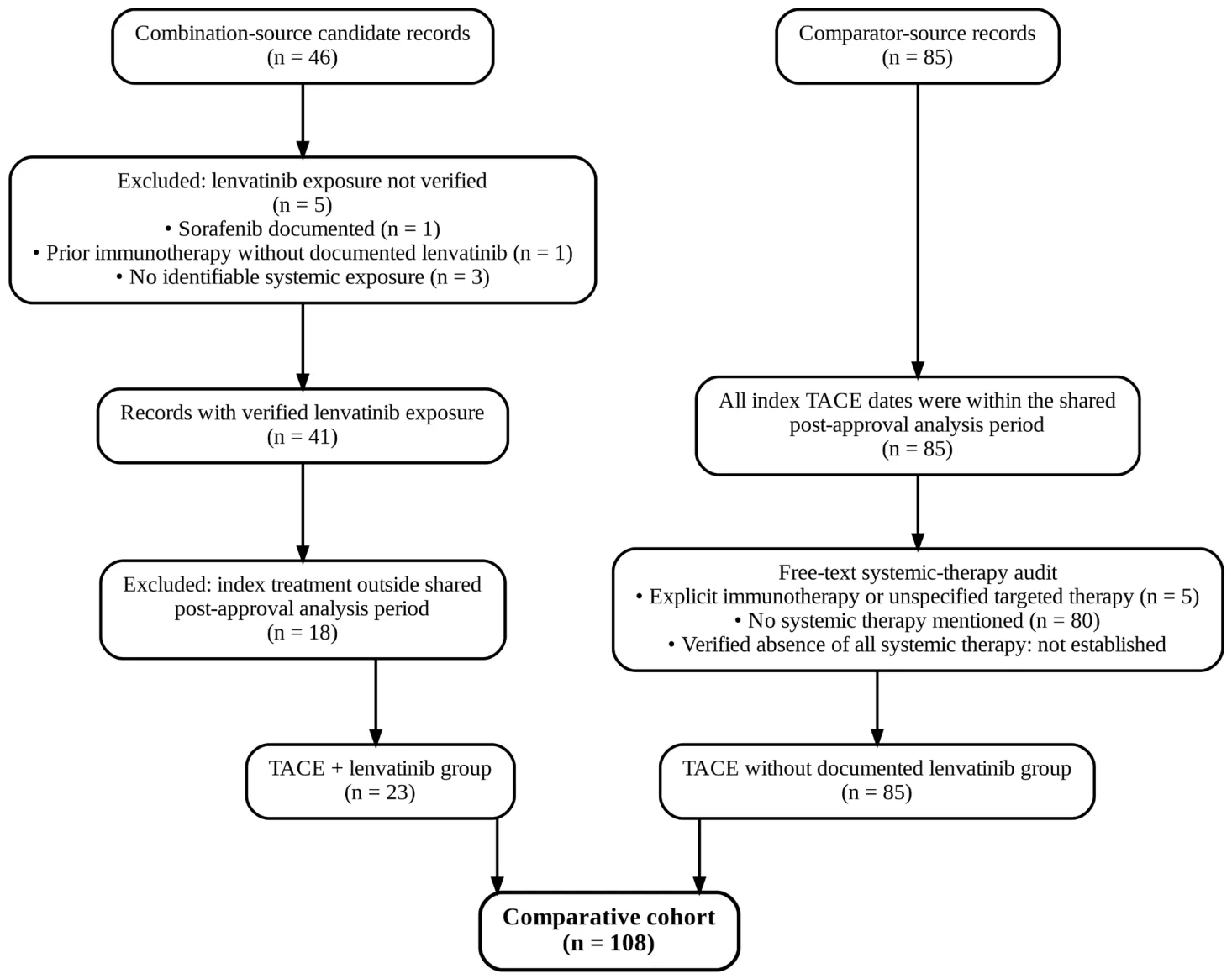
Record inclusion and exposure classification for the shared post-approval analysis period. The comparator label denotes absence of documented lenvatinib, not verified TACE monotherapy. TACE, transarterial chemoembolization.

Of 46 combination-source candidate records, 5 were excluded because lenvatinib exposure could not be verified and 18 were outside the shared post-approval period, leaving 23 patients. All 85 comparator-source records had index TACE dates within the analysis period. Free-text audit identified five comparator records with explicit documentation of systemic anticancer treatment not identified as lenvatinib (one mentioning immunotherapy and four mentioning unspecified targeted therapy); 80 had no systemic-therapy mention, but verified absence of all systemic therapy could not be established. The comparative cohort comprised 108 patients (Figure 1).

Age differed little between groups, but several clinical and procedural variables were imbalanced. PVTT was recorded in 2/23 combination patients and 0/85 comparator patients (ASD, 0.44). Conventional TACE was used in 47.8% versus 81.2% (ASD, 0.74), whereas hybrid/combined TACE was recorded in 30.4% versus 1.2% (ASD, 0.88). ALBI grade 1 also differed among available cases (55.6% versus 34.0%; ASD, 0.44). Baseline prognostic fields were substantially less complete in the comparator source; for example, viral-hepatitis and AFP fields were missing in 36/85 comparator records but only 1/23 combination records (Table 1).

### 3.2. Recorded Imaging Response and Missing-Outcome Sensitivity

A RECIST v1.1 CR or PR was recorded for 17/23 (73.9%) combination patients and 30/85 (35.3%) comparator patients. The absolute recorded-response difference was 38.6 percentage points (95% CI, 18.0–59.2), the RR was 2.09 (95% CI, 1.44–3.05), and the OR was 5.19 (95% CI, 1.85–14.57). These estimates describe the probability that a response was both present in the source category and recorded; they do not identify the biological response probability when outcomes are missing.

**Figure 2:**
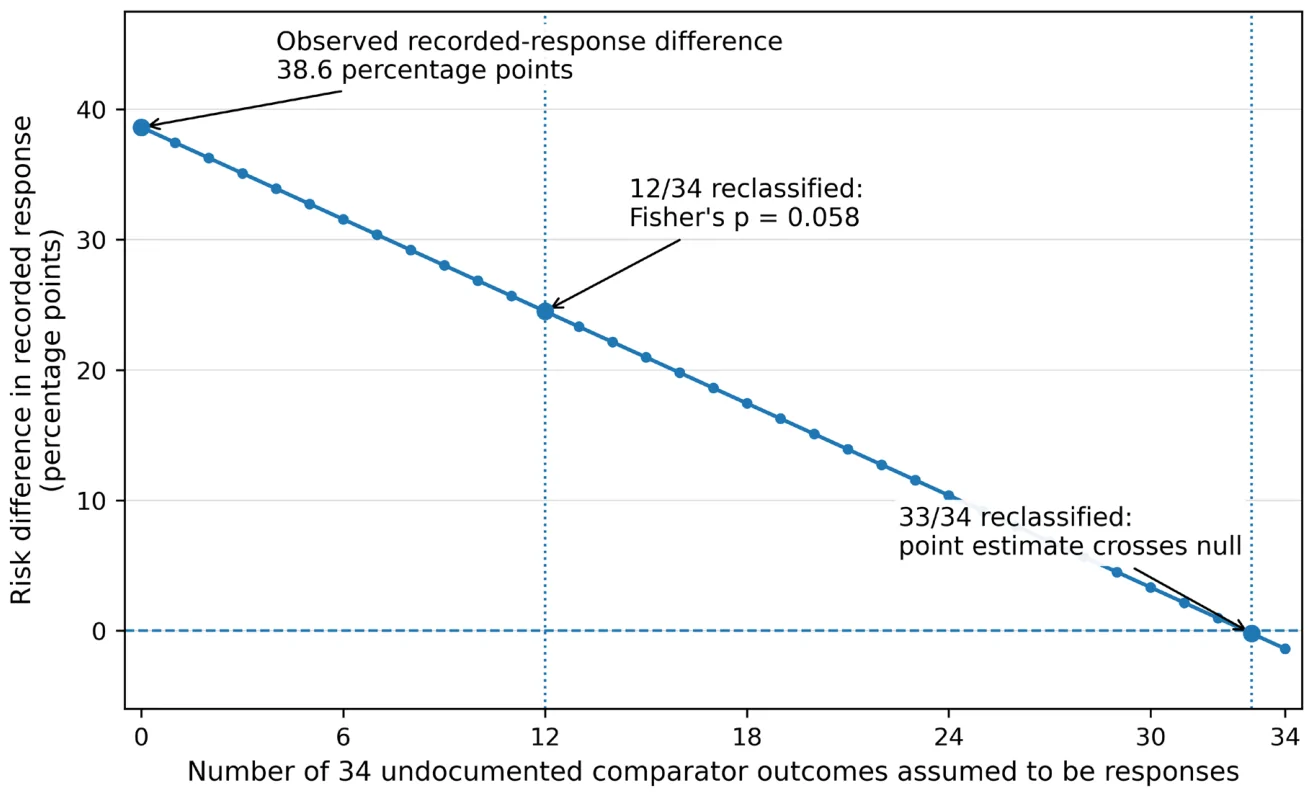
Tipping-point analysis for outcome missingness. All six undocumented combination outcomes were held as nonresponses, while 0–34 undocumented comparator outcomes were sequentially treated as responses. The observed recorded-response difference was 38.6 percentage points; 12 reclassifications made the two-sided Fisher’s exact *p*-value exceed 0.05, and 33 eliminated the point-estimate advantage. The plot demonstrates sensitivity to missing-outcome assumptions rather than a causal treatment effect.

Outcome status was undocumented for 6/23 (26.1%) combination patients and 34/85 (40.0%) comparator patients. Allowing every undocumented outcome to be either a response or a nonresponse produced nonparametric response-rate bounds of 73.9–100.0% for the combination group and 35.3–75.3% for the comparator group. The corresponding RD bounds were −1.4 to 64.7 percentage points and RR bounds were 0.98 to 2.83. In the tipping-point sequence that held all six undocumented combination outcomes as nonresponses, reclassifying 12 of 34 undocumented comparator outcomes as responses made the two-sided Fisher’s exact *p*-value exceed 0.05 (recorded-response proportions 73.9% versus 49.4%; OR, 2.90; *p* = 0.058). Reclassifying 13 made the Wald OR confidence interval include 1, and reclassifying 33 eliminated the point-estimate advantage (73.9% versus 74.1%; RD, −0.2 percentage points; OR, 0.99). The adverse extreme case with all 34 comparator outcomes treated as responses yielded an OR of 0.93 (95% CI, 0.32–2.67) (Table 2; Figure 2).

### 3.3. Limited Adjustment and Exposure-Classification Sensitivity

Within the limited covariate sets that could be fitted, the age- and treatment-year-adjusted OR was 5.07 (95% CI, 1.80–14.30), and additional adjustment for initial TACE technique yielded an OR of 4.50 (95% CI, 1.52–13.34). These models do not control the full treatment-selection process identified in the causal framework. The expanded complete-case model—including age, year, TACE technique, AFP, and continuous ALBI score—contained 63 patients and was attenuated and imprecise (adjusted OR, 1.97; 95% CI, 0.45–8.60). The change in estimate indicates model-population and selection sensitivity rather than successful adjustment for all important confounders.

The comparator audit identified five records with explicit non-lenvatinib systemic co-therapy. Excluding these records yielded recorded responses in 17/23 (73.9%) versus 27/80 (33.8%): RD, 40.2 percentage points (95% CI, 19.4–60.9); RR, 2.19 (95% CI, 1.48–3.24); unadjusted OR, 5.56 (95% CI, 1.97–15.73); and age- and year-adjusted OR, 5.49 (95% CI, 1.93–15.61). This restriction addresses identifiable co-therapy only; it does not verify TACE monotherapy among the remaining comparator records (Table 3; Supplementary Figure S2).

### 3.4. Response Availability and Covariate Co-Missingness

A response category was recorded for 68 patients and unavailable for 40. The treatment-group composition (25.0% versus 15.0% combination; ASD, 0.25) and TACE-technique distribution differed between response-available and response-unavailable records. More importantly, baseline-data availability differed by 80–100 percentage points for several prognostic variables: viral-hepatitis status was available in 97.1% versus 12.5%, AFP in 95.6% versus 15.0%, tumor-burden score in 100.0% versus 2.5%, maximum tumor diameter in 100.0% versus 0%, and ALBI score in 94.1% versus 10.0%. This structured co-missingness means that complete-case models selected a clinically and procedurally different subset rather than merely reducing sample size (Table 4).

### 3.5. Treatment Timeline, Safety, and Survival Ascertainment

The median number of documented TACE sessions was 2 (IQR, 1–3) in the combination group and 1 (IQR, 1–2) in the comparator group. Exact lenvatinib initiation, continuation, interruption, repeat TACE before first imaging, and index-to-imaging intervals were not uniformly retained. Therefore, a standardized response window or patient-level treatment timeline could not be reconstructed, and response cannot be attributed solely to the index TACE.

Postembolization syndrome was entered for 17/23 combination and 50/85 comparator patients; every completed field indicated the syndrome, showing that the field could not support incidence estimation. Grade 1 hand–foot skin reaction was recorded in 4/23 combination patients. The database contained no explicit grade ≥3 lenvatinib-related-event or treatment-related-death entry, but surveillance was incomplete and non-harmonized. These are statements about documentation, not confirmed absence of events; safety incidence was not estimable. Progression dates were available for 5/23 versus 44/85 patients, and death dates for 3/23 versus 24/85. Comparative safety, progression-free survival, and overall survival were therefore not reported (Table 5).

## 4. Discussion

In this Vietnamese institutional cohort, a RECIST v1.1 CR or PR was recorded more often after the TACE-plus-lenvatinib strategy than in records without documented lenvatinib. The observed RD was 38.6 percentage points and the RR was 2.09. These values describe recorded responses under the available documentation process; they do not establish a causal comparative treatment effect. The key result of the revision is that identification is weak: worst-case bounds included no difference, 12 of 34 undocumented comparator outcomes reclassified as responses removed conventional Fisher’s significance, and 33 eliminated the point-estimate advantage. The expanded complete-case estimate was also substantially attenuated and imprecise.

Outcome missingness is therefore the central inferential issue rather than a secondary sensitivity analysis. Response availability was linked to profound co-missingness of AFP, tumor burden, tumor diameter, and ALBI. This pattern is inconsistent with treating missingness as an isolated random omission. The response-evaluable analysis selects patients who reached, accessed, and had documented imaging and may therefore be conditional on prognosis, clinician practice, treatment continuation, or the response itself. Likewise, complete-case regression selects a different subset and can introduce selection bias. Multiple imputation would not solve this problem without defensible auxiliary variables and a credible missing-at-random model; transparent bounds more faithfully display the uncertainty supported by these data.

The endpoint is also narrower than conventional clinical-trial ORR. Imaging timing, reader identity, blinding, and adjudication were not uniformly known, SD was not explicitly represented, and source images were not centrally reread. RECIST v1.1 does not directly characterize viable enhancing tumor after locoregional HCC therapy as mRECIST does [4,5,19]. The 73.9% versus 35.3% comparison may therefore reflect differences in tumor biology, imaging opportunity, timing, interpretation, or documentation. We consequently relabeled the endpoint throughout as a documented RECIST v1.1 response at first recorded imaging and do not equate it with a standardized centrally adjudicated ORR.

Exposure classification was asymmetric. Lenvatinib was positively verified in the combination group, whereas the comparator was defined by absence of a lenvatinib mention. Five comparator records explicitly mentioned other systemic therapy; excluding them did not materially change the observed association. Nevertheless, the remaining 80 records cannot be assumed to represent true TACE monotherapy because complete medication histories, undocumented lenvatinib, other systemic agents, and timing relative to imaging were not verifiable. The restricted analysis therefore addresses identifiable co-therapy but cannot eliminate exposure misclassification or time-varying co-intervention bias.

Confounding by indication is also plausible. The combination group contained all recorded PVTT cases, used hybrid/combined TACE far more often, and had more complete baseline documentation. These differences suggest selection according to disease extent, procedural strategy, multidisciplinary judgment, anticipated tolerance, or access rather than random allocation. Adjustment for age, year, and a binary TACE-technique term is conditional on a limited covariate set and should not be interpreted as robust control of treatment selection. PVTT could not be reliably modeled because only two events occurred, and technique-specific outcome estimates would be dominated by sparse cells—particularly seven-versus-one hybrid cases—and differential outcome missingness. The causal framework makes these limitations explicit instead of implying that regression resolves them.

The VEGF/FGF rationale remains biologically plausible but is not empirical proof that the observed magnitude is pharmacologic. A more discriminating mechanistic prediction is that antiangiogenic benefit should be greatest when TACE leaves viable, highly vascular, incompletely embolized tumor; related phenotypes might include macrovascular invasion or higher burden when hepatic reserve permits combined therapy. Prospective studies could test this prediction using prespecified mRECIST viable-tumor measurements, angiographic selectivity, vascularity, embolization completeness, exposure duration, and interaction analyses. The present data cannot distinguish that mechanism from treatment intensity, patient selection, or imaging-process differences.

The principal contribution is therefore not a definitive estimate of comparative effectiveness. It is a Vietnamese real-world description showing a clinically notable recorded-response difference together with specific implementation and registry gaps: source-specific baseline completeness, incomplete medication reconciliation, unstandardized early imaging, uncertain treatment timelines, non-harmonized safety fields, and insufficient event-date capture. These findings identify what must be standardized before local comparative effectiveness can be estimated credibly and provide a transparent hypothesis for a prospective study.

Even fewer safety and survival findings are identified. A database containing no explicit grade ≥3 event entry does not demonstrate absence of severe toxicity when surveillance fields are incomplete. Similarly, sparse and differential progression, death, and censoring dates invalidate routine Kaplan–Meier or Cox comparisons. The appropriate conclusion is that safety incidence and survival were not estimable, not that the strategies were similarly safe or that severe events did not occur.

The strengths of this study include restriction to a shared post-approval period, positive verification of combination exposure, manual audit of comparator treatment text, explicit separation of baseline and outcome missingness, reporting of absolute and relative effect measures, nonparametric bounds, a quantitative tipping-point analysis, and an explicit causal and measurement framework. The limitations include the single-center nonrandomized design, database-defined sampling frame, small combination group, sparse PVTT and technique strata, incomplete eligibility verification, differential missingness, uncertain treatment and imaging timelines, lack of central mRECIST review, asymmetric exposure classification, residual confounding, and inability to estimate safety or survival. The findings should be regarded as hypothesis-generating and as evidence for registry improvement.

## 5. Conclusions

Recorded RECIST v1.1 complete or partial responses at first documented imaging were more frequent after the TACE-plus-lenvatinib strategy than in records without documented lenvatinib. However, nonparametric bounds spanned the null, modest reclassification of undocumented comparator outcomes changed inferential conclusions, and complete-case estimates were unstable. Imaging ascertainment, treatment-selection confounding, incompletely characterized comparator therapy, and uncertain treatment timelines prevent identification of a comparative treatment effect. The study primarily identifies Vietnamese registry and implementation gaps and supports a prospective, independently reviewed mRECIST study with complete treatment, safety, and survival ascertainment.

## Figures and Tables

**Table 1:** Baseline characteristics, descriptive balance, and source-data completeness.

Characteristic	TACE + Lenvatinib (*n* = 23)	TACE Without Documented Lenvatinib (*n* = 85)	Descriptive Balance	Missing, Combination/Comparator
Age, years, median (IQR)	59.0 (50.5–69.0)	62.0 (55.0–68.0)	Median difference −3.0 years	0/0
Viral hepatitis, *n/N* available (%)	22/22 (100.0)	46/49 (93.9)	ASD 0.36	1 (4.3%)/36 (42.4%)
AFP > 400 ng/mL, *n/N* available (%)	12/22 (54.5)	22/49 (44.9)	ASD 0.19	1 (4.3%)/36 (42.4%)
Tumor-burden score > 7, *n/N* available (%)	9/17 (52.9)	26/52 (50.0)	ASD 0.06	6 (26.1%)/33 (38.8%)
Largest tumor diameter, cm, median (IQR)	9.0 (7.2–10.6); *n* = 17	8.8 (7.5–11.0); *n* = 51	Median difference +0.2 cm	6 (26.1%)/34 (40.0%)
ALBI grade 1, *n/N* available (%)	10/18 (55.6)	17/50 (34.0)	ASD 0.44	5 (21.7%)/35 (41.2%)
Portal vein tumor thrombosis, *n* (%)	2 (8.7)	0 (0.0)	ASD 0.44	0/0
Index treatment in 2025, *n* (%)	14 (60.9)	50 (58.8)	ASD 0.04	0/0
Conventional TACE, *n* (%)	11 (47.8)	69 (81.2)	ASD 0.74	0/0
Drug-eluting bead TACE, *n* (%)	5 (21.7)	12 (14.1)	ASD 0.20	0/0
Hybrid/combined TACE, *n* (%)	7 (30.4)	1 (1.2)	ASD 0.88	0/0
TACE technique not documented, *n* (%)	0 (0.0)	3 (3.5)	ASD 0.27	0/3 (3.5%)

ASD denotes absolute standardized difference for binary variables and is descriptive rather than a hypothesis test; values > 0.10 flag potential imbalance. Continuous variables are summarized by median difference because means and standard deviations were unavailable. ASDs can be unstable when proportions approach 0 or 1 and should be interpreted together with numerators, denominators, and missingness. “Tumor-burden score > 7” is reproduced as recorded; component-level data were insufficient for independent reconstruction. AFP, alpha-fetoprotein; ALBI, albumin–bilirubin; IQR, interquartile range; TACE, transarterial chemoembolization.

**Table 2:** Recorded RECIST v1.1 response and missing-outcome sensitivity analyses.

Outcome or Scenario	TACE + Lenvatinib	TACE Without Documented Lenvatinib	Effect Estimate/Interpretation
Complete response	1/23 (4.3%)	0/85 (0.0%)	—
Partial response	16/23 (69.6%)	30/85 (35.3%)	—
Progressive disease (PD)	0/23 (0.0%)	21/85 (24.7%)	—
Stable disease	Not explicitly represented	Not explicitly represented	Not estimable
Response undocumented	6/23 (26.1%)	34/85 (40.0%)	Outcome unknown
Recorded CR/PR among all records	17/23 (73.9%)	30/85 (35.3%)	RD 38.6 pp (95% CI, 18.0–59.2); RR 2.09 (95% CI, 1.44–3.05); OR 5.19 (95% CI, 1.85–14.57)
Nonparametric response-rate bounds	73.9–100.0%	35.3–75.3%	RD bounds −1.4 to 64.7 pp; RR bounds 0.98–2.83
Tipping point for Fisher’s *p* ≥ 0.05: 12/34 comparator unknowns reclassified as responses	17/23 (73.9%)	42/85 (49.4%)	RD 24.5 pp; RR 1.50; OR 2.90; *p* = 0.058
Tipping point for Wald OR CI to include 1: 13/34 reclassified	17/23 (73.9%)	43/85 (50.6%)	OR 2.77 (95% CI, 0.99–7.70)
Point estimate null: 33/34 reclassified	17/23 (73.9%)	63/85 (74.1%)	RD −0.2 pp; RR 1.00; OR 0.99
Adverse extreme case: 34/34 reclassified	17/23 (73.9%)	64/85 (75.3%)	RD −1.4 pp; RR 0.98; OR 0.93 (95% CI, 0.32–2.67)
Response-evaluable selected subset	17/17 (100.0%)	30/51 (58.8%)	RR 1.70 (95% CI, 1.35–2.14); vulnerable to selection bias

The observed numerator is the number of records with a documented CR or PR. Undocumented outcomes are unknown, not confirmed nonresponses. In the tipping-point sequence, all six undocumented combination outcomes were held as nonresponses, while comparator outcomes were reclassified sequentially. pp, percentage points; CI, confidence interval; CR, complete response; OR, odds ratio; PD, progressive disease; PR, partial response; RD, risk difference; RR, risk ratio. RECIST, Response Evaluation Criteria in Solid Tumors.

**Table 3:** Regression and exposure-classification sensitivity analyses.

Analysis	*n*	Estimate	Interpretation
Age and treatment year	108	aOR 5.07 (95% CI, 1.80–14.30)	Limited confounder set
Age, year, and initial TACE technique	108	aOR 4.50 (95% CI, 1.52–13.34)	Does not address unmeasured tumor extent, hepatic reserve, performance status, or prior therapy
Expanded complete case: age, year, TACE technique, AFP > 400 ng/mL, continuous ALBI	63	aOR 1.97 (95% CI, 0.45–8.60)	Selected subset; attenuated and imprecise
Exclude 5 comparator records with explicit systemic co-therapy	103	RD 40.2 pp (95% CI, 19.4–60.9); RR 2.19 (95% CI, 1.48–3.24); OR 5.56 (95% CI, 1.97–15.73)	Remaining comparator exposure still incompletely characterized
Same restricted cohort, adjusted for age and treatment year	103	aOR 5.49 (95% CI, 1.93–15.61)	Conditional on a limited confounder set

Adjusted models used logistic regression. These analyses are descriptive and do not identify a causal effect because important treatment-selection variables were incompletely observed. AFP, alpha-fetoprotein; ALBI, albumin–bilirubin; aOR, adjusted odds ratio; CI, confidence interval; OR, odds ratio; pp, percentage points; RD, risk difference; RR, risk ratio; TACE, transarterial chemoembolization.

**Table 4:** Characteristics and data completeness according to response availability.

Characteristic	Response Recorded (*n* = 68)	Response not Recorded (*n* = 40)	Descriptive Contrast
TACE + lenvatinib, *n* (%)	17 (25.0)	6 (15.0)	ASD 0.25
Age, years, median (IQR)	60.0 (54.8–67.3)	62.5 (56.0–69.0)	Median difference −2.5 years
Index treatment in 2025, *n* (%)	42 (61.8)	22 (55.0)	ASD 0.14
Conventional TACE, *n* (%)	46 (67.6)	34 (85.0)	ASD 0.42
Drug-eluting bead TACE, *n* (%)	12 (17.6)	5 (12.5)	ASD 0.14
Hybrid/combined TACE, *n* (%)	7 (10.3)	1 (2.5)	ASD 0.32
PVTT, *n* (%)	2 (2.9)	0 (0.0)	ASD 0.25
Viral-hepatitis field available, *n* (%)	66 (97.1)	5 (12.5)	Completeness difference +84.6 pp
AFP field available, *n* (%)	65 (95.6)	6 (15.0)	Completeness difference +80.6 pp
Tumor-burden score available, *n* (%)	68 (100.0)	1 (2.5)	Completeness difference +97.5 pp
Maximum tumor diameter available, *n* (%)	68 (100.0)	0 (0.0)	Completeness difference +100.0 pp
ALBI score available, *n* (%)	64 (94.1)	4 (10.0)	Completeness difference +84.1 pp
Explicit systemic co-therapy in comparator free text, *n/N* comparator (%)	5/51 (9.8)	0/34 (0.0)	Documentation-dependent; not a verified absence comparison

Response availability required a recorded CR, PR, SD, or PD. ASDs are descriptive. Clinical values for incompletely observed covariates were not compared because only 0–6 response-unavailable records had data, making available-case contrasts unstable. AFP, alpha-fetoprotein; ALBI, albumin–bilirubin; ASD, absolute standardized difference; IQR, interquartile range; pp, percentage points; PVTT, portal vein tumor thrombosis; TACE, transarterial chemoembolization.

**Table 5:** Treatment-timeline, safety, and survival ascertainment.

Measure	TACE + Lenvatinib (*n* = 23)	TACE Without Documented Lenvatinib (*n* = 85)	Interpretation
Index TACE date	23/23 available	85/85 available	Used to anchor inclusion
Verified lenvatinib exposure	23/23	0/85 by definition	Positively verified only in combination group
Explicit non-lenvatinib systemic co-therapy	0 identified in combination records used	5/85	One immunotherapy mention; four unspecified targeted-therapy mentions
Verified absence of all systemic therapy	Not applicable	Not established	Comparator is not confirmed TACE monotherapy
Documented TACE sessions, median (IQR)	2 (1–3)	1 (1–2)	Treatment intensity may differ before imaging
Exact lenvatinib start/continuation/interruption	Not uniformly retained	Not applicable	No patient-level exposure-duration analysis
Repeat TACE before first recorded imaging	Not uniformly retained	Not uniformly retained	Response cannot be isolated to index TACE
Exact index-to-imaging interval	Not uniformly retained	Not uniformly retained	No standardized-window sensitivity analysis possible
Postembolization-syndrome field completed	17/23	50/85	Field missing for 6 and 35; incidence not estimable
Grade 1 hand–foot skin reaction entry	4/23	Not systematically captured	Descriptive entry only
Grade ≥3 lenvatinib-related-event entry	None recorded	Not applicable	No event entry does not establish absence
Treatment-related-death entry	None recorded	None recorded	Safety surveillance incomplete
Progression date available	5/23 (21.7%)	44/85 (51.8%)	Differential completeness
Death date available	3/23 (13.0%)	24/85 (28.2%)	Differential completeness

“None recorded” means that no event entry was found in the available fields; it does not mean that the event was confirmed to be absent. Adverse-event incidence and comparative safety were not estimable. IQR, interquartile range; TACE, transarterial chemoembolization.

## Data Availability

The data presented in this study are available from the corresponding author upon reasonable request and subject to institutional and ethics approval. The data are not publicly available because the source clinical databases contain protected health information.

## Supplementary Materials

The following supporting information can be downloaded at: https://www.mdpi.com/article/10.3390/jcm15176839/s1, Figure S1: Causal and measurement framework for treatment selection, data availability, and recorded imaging response; Figure S2: Regression and exposure-classification sensitivity estimates.
