# Supplementary material for: Recorded Early Imaging Responses After Transarterial Chemoembolization Plus Lenvatinib Versus Transarterial Chemoembolization Without Documented Lenvatinib for Unresectable Hepatocellular Carcinoma: A Vietnamese Cohort Study
Source: J Clin Med. 2026 Sep 3;15(17):6839. doi: 10.3390/jcm15176839 (PMC13565755; doi:10.3390/jcm15176839)
Supplement: Supplementary file 1 [file jcm-15-06839-s001.zip › jcm-4520464-supplementary.pdf]

Supplementary

Recorded Early Imaging Response after Transarterial Chemoembolization Plus Lenvatinib versus Transarterial Chemoembolization without Documented Lenvatinib for Unresectable Hepatocellular Carcinoma: A Vietnamese Cohort Study

Vu Le Minh et al.

Supplementary Figure S1

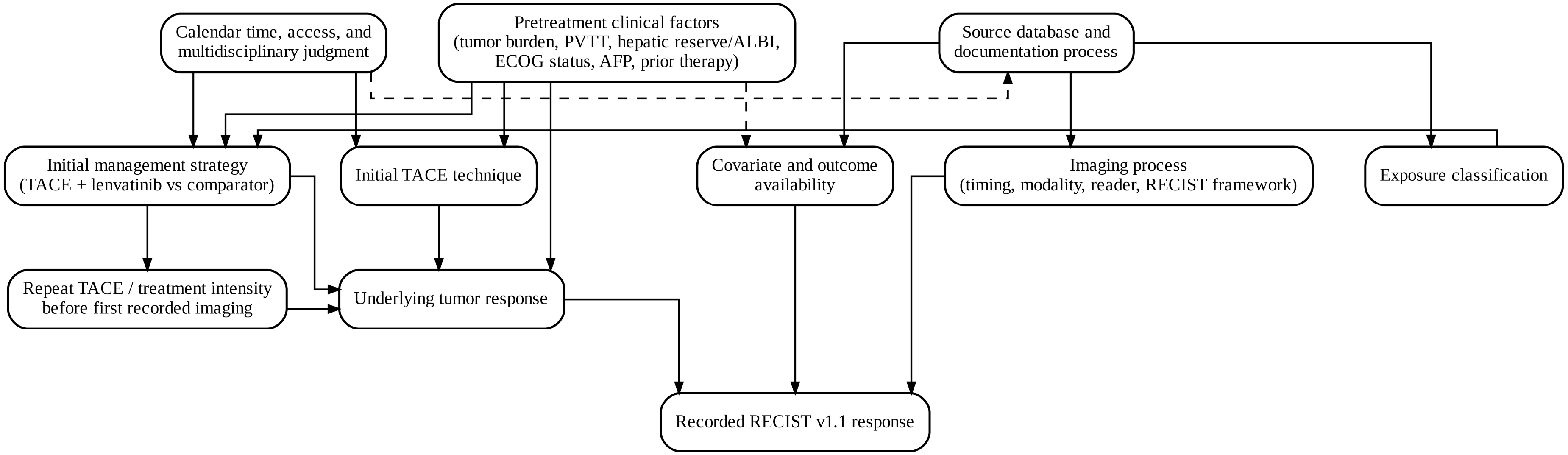

Figure S1. Causal and measurement framework. Solid arrows denote clinically or procedurally plausible pathways linking pretreatment factors, treatment selection, TACE technique, repeat treatment, imaging, and recorded response. Dashed arrows emphasize source-dependent documentation and co-missingness. The framework distinguishes underlying tumor response from the response category recorded in the database. AFP, alpha-fetoprotein; ALBI, albumin–bilirubin; ECOG, Eastern Cooperative Oncology Group; PVTT, portal vein tumor thrombosis; RECIST, Response Evaluation Criteria in Solid Tumors; TACE, transarterial chemoembolization.

Supplementary Figure S2

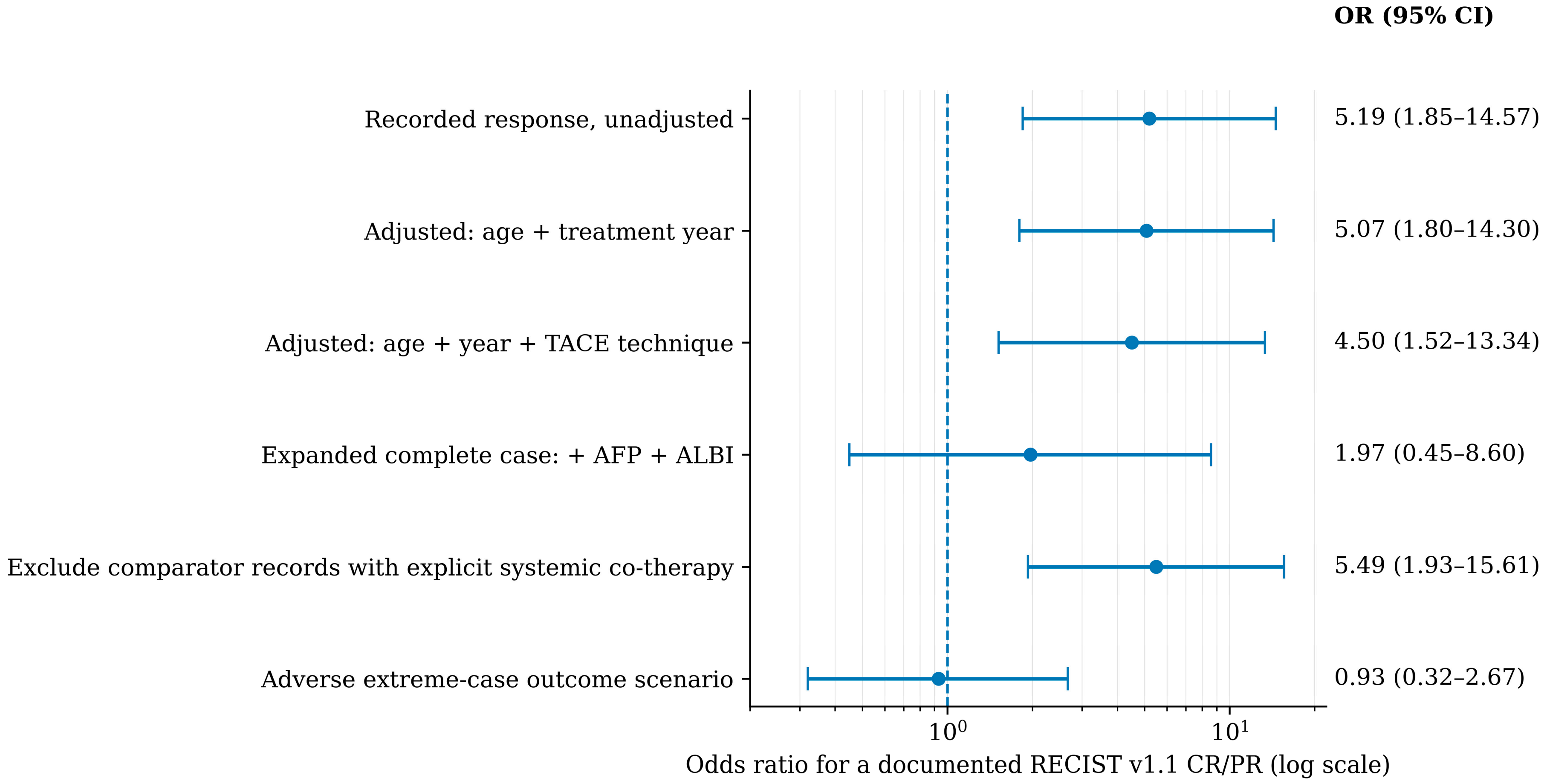

Figure S2. Regression and exposure-classification sensitivity estimates. Points indicate odds ratios and horizontal lines indicate 95% confidence intervals. The models are descriptive and conditional on the covariates observed; they do not constitute complete control of confounding. The adverse extreme-case scenario treats all undocumented combination outcomes as nonresponses and all undocumented comparator outcomes as responses. AFP, alpha-fetoprotein; ALBI, albumin–bilirubin; CI, confidence interval; OR, odds ratio; TACE, transarterial chemoembolization.
